# Genetic and Epigenetic Risks of Male Infertility in ART

**DOI:** 10.3390/ijms262411812

**Published:** 2025-12-07

**Authors:** Athanasios Zikopoulos, Periklis Katopodis, Maria Filiponi, Athanasios Zachariou, Athanasia Sesse, Ioanna Bouba, Charilaos Kostoulas, Sofia Markoula, Ioannis Georgiou

**Affiliations:** 1 Royal Cornwall Hospitals NHS Treliske Truro, Foundation Trust U.K., Truno TR1 3LJ, UK; thanzik92@gmail.com; 2 Laboratory of Medical Genetics in Clinical Practice, Faculty of Medicine, School of Health Sciences, University of Ioannina, 45110 Ioannina, Greece; katopodisper@gmail.com (P.K.); a.sesse@uoi.gr (A.S.); ibouba@uoi.gr (I.B.); chkost@uoi.gr (C.K.); 3 Urology Outpatient Department, Kentavros Physical Medicine and Rehabilitation Centre, 38222 Volos, Greece; mfilipou@gmail.com; 4 Department of Urology, Faculty of Medicine, School of Health Sciences, University of Ioannina, 45110 Ioannina, Greece; zahariou@otenet.com; 5 Department of Neurology, Faculty of Medicine, School of Health Sciences, University of Ioannina, 45110 Ioannina, Greece; smarkoula@uoi.gr

**Keywords:** genetic risks, epigenetic risks, intracytoplasmic sperm injection, testis, male infertility

## Abstract

Assisted reproductive technologies (ART) and, in particular, intracytoplasmic sperm injection (ICSI) transformed the management of male infertility by making fertility possible in previously untreatable cases. However, the bypassing of natural selection mechanisms at fertilization is fraught with the danger of transmission of genetic and epigenetic abnormalities. Male infertility is now a multifactorial disorder with notable contributions from single-gene defects, chromosomal abnormalities, and Y-chromosome microdeletions. The recent advances in next-generation sequencing and sperm omics have identified mutations and copy-number variations in genes critical for spermatogenesis, flagellar structure, and endocrine regulation. Along with these findings, an increasing body of evidence suggests that ART procedures can lead to a disruption of epigenetic reprogramming during gametogenesis and early embryogenesis, resulting in imprinting disorders and altered patterns of gene expression in the offspring. This review synthesizes recent progress in the molecular underpinnings of genetic and epigenetic hazards of ART, with an emphasis on clinical significance for reproductive counselling and ethical considerations for future generations.

## 1. Introduction

Infertility occurs in about 15% of couples of reproductive age across the globe, and male factors are responsible for about half of these [1]. The advent of assisted reproductive technologies, more importantly intracytoplasmic sperm injection (ICSI), has transformed treatment options for severe male-factor infertility. Through fertilization with a single spermatozoon, ICSI overcomes a number of physiological hurdles such as sperm selection and natural defence mechanisms of the oocyte. Although this approach has dramatically increased pregnancy rates, it has also solicited concern regarding the potential transmission of genetic and epigenetic abnormalities carried in the paternal germline [2,3].

Male infertility encompasses a heterogeneous assortment of aetiologies, ranging from single-gene defects and chromosomal abnormalities to complex polygenic and environmental interactions [4]. Up to 30% of cases are idiopathic, often with subtle genomic or epigenomic dysregulation that is not detectable with conventional testing [5]. Whole-genome and whole-exome sequencing genomic medicine advances have uncovered a growing list of genes implicated in spermatogenic failure -such as defects in spermatogonial proliferation, meiosis, or spermatogenesis-, sperm motility disorders, and endocrine regulation of testicular function. The integration of these findings with transcriptomic and epigenomic research has demonstrated that genetic and epigenetic causes of male infertility are interconnected and often co-regulated [6,7].

Apart from genetic aetiology, recent evidence points to the role of epigenetic mechanisms (DNA methylation, histone modification, and small noncoding RNAs) in the regulation of sperm function and embryonic development [8,9,10,11]. Exposures to the environment, paternal age, obesity, and oxidative stress are all linked to epigenetic alterations in spermatozoa that can be trans-generationally heritable. The process of assisted reproduction itself can also play a role through in vitro manipulation, cryopreservation, and culture media. As such, assisted reproductive technologies (ART) offspring do have a marginally increased frequency of imprinting disorders and epigenetic dysregulation, though the absolute risk is low [12,13,14].

The aim of this review is to provide an update on the genetic and epigenetic mechanisms contributing to male infertility in the context of ART. Focusing mostly on data from the past twenty years, this review highlights the mechanisms of heritable and acquired defects of spermatogenesis, the impact of ART on germline integrity, and the implications for reproductive counselling, screening, and long-term offspring health.

## 2. Genetic Basis of Male Infertility

### 2.1. Overview of Genetic Contributions to Male Infertility

**Figure 1:**
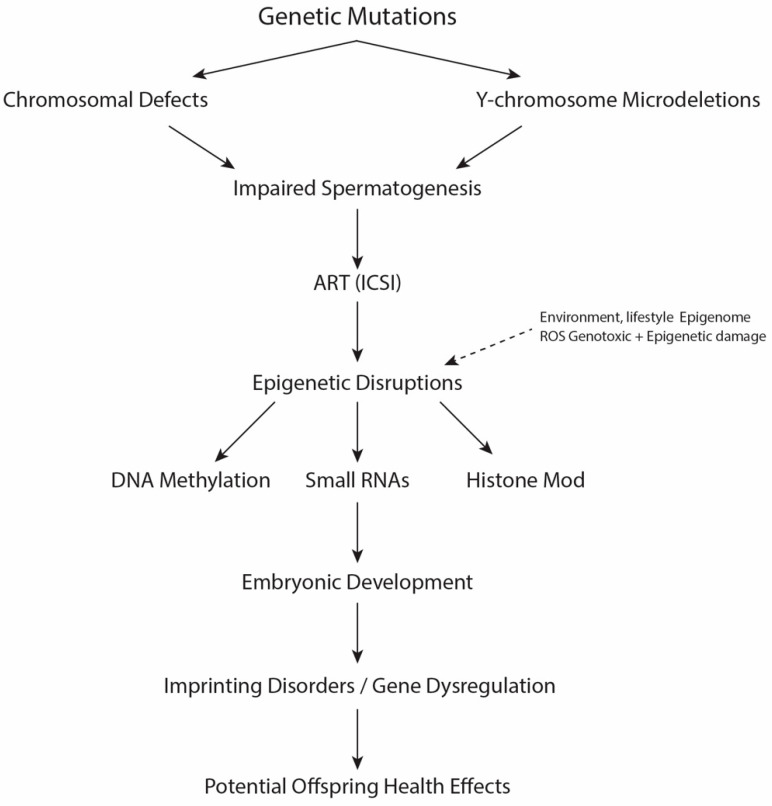
Mechanistic Pathways of Genetic and Epigenetic Risk Transmission in Male Infertility and ART. Overview of the molecular mechanisms linking genetic and epigenetic defects in male infertility and their transmission through ART. Single-gene mutations, chromosomal abnormalities, and Y-chromosome microdeletions impair spermatogenesis. The use of ART, particularly ICSI, bypasses natural gamete selection, allowing sperm with compromised genomic or epigenomic integrity to fertilize oocytes. Environmental factors and oxidative stress exacerbate epigenetic dysregulation. These molecular disruptions may affect embryonic development, imprinting, and offspring health.

Male infertility is a complex disorder with monogenic, chromosomal, and polygenic aetiology that has long been recognized (Figure 1). Early research estimated that 15–30% of male infertility is accounted for by recognized genetic factors; however, emerging multi-omics research demonstrates that the proportion may be much higher [15]. Genomic technologies revealed that a significant proportion of male infertility cases that were previously idiopathic are underpinned by mutations in genes that regulate spermatogonial proliferation, meiosis, or sperm structural integrity. They include genes that regulate flagellar assembly (Dynein Axonemal Heavy Chain 1—*DNAH1*, Cilia and Flagella Associated Protein 43—*CFAP43*, Cilia and Flagella Associated Protein 44—*CFAP44*), meiosis (Testis Expressed 11—*TEX11*, Synaptonemal Complex Protein 3—*SYCP3*), and chromatin remodelling (Transition Protein 1—*TNP1*, Protamine 2—*PRM2*). Use of next-generation sequencing (NGS) panels now enables high-resolution genetic characterization of infertile men, with diagnostic yields of up to 40% in non-obstructive azoospermia (NOA) and severe oligozoospermia [6,16,17].

Genetic defects found in infertile men often extend beyond classical chromosomal abnormalities. Copy number variations (CNVs), small insertions or deletions, and point mutations of important genes on autosomes and sex chromosomes have been described [5]. The increasing availability of population-level reference datasets, such as gnomAD, has facilitated variant interpretation and genotype-phenotype correlation. Functional validation through animal models and patient-derived induced pluripotent stem cells has also delineated causal links between gene defects and spermatogenic arrest. Accumulating evidence highlights that infertility itself may be a sentinel phenotype for generalized genomic instability and predisposition to somatic disease, such as cancer and metabolic dysfunctions [18].

### 2.2. Single-Gene Disorders and Monogenic Infertility

#### 2.2.1. Cystic Fibrosis Transmembrane Conductance Regulator (CFTR) Mutations and Congenital Bilateral Absence of the Vas Deferens (CBAVD)

*CFTR* gene mutations remain the most frequent monogenic origin of obstructive azoospermia, leading to congenital bilateral absence of the vas deferens (CBAVD). The prevalence of *CFTR* mutations among CBAVD patients is 60–90%, and compound heterozygosity for severe and mild alleles will result in partial or complete obstruction of the excurrent ducts [19].

High-throughput sequencing, since 2006, has expanded the inventory of *CFTR* variants, identifying over 2000 alleles with variable penetrance. Polymorphisms in intron 8 (5T, 7T, 9T repeats) and mutations such as R117H modulate splicing efficiency and protein function, influencing the clinical phenotype. Current recommendations include *CFTR* mutation screening and genetic counselling for all men with obstructive azoospermia prior to ART, with preimplantation genetic testing (PGT) when both partners are carriers of pathogenic variants [20,21,22].

#### 2.2.2. Ciliary Dyskinesia and Flagellar Disorders

Primary ciliary dyskinesia (PCD), including Kartagener syndrome, is caused by mutations in genes encoding axonemal dynein arms and radial spoke proteins. Since the initial descriptions of Dynein Axonemal Heavy Chain 5 (DNAH5) and Dynein Axonemal Intermediate Chain 1 (DNAI1), over 40 causative genes have now been described, including Coiled-Coil Domain 39 Molecular Ruler Complex Subunit (*CCDC39*), Coiled-Coil Domain 40 Molecular Ruler Complex Subunit (*CCDC40*), Dynein Axonemal Heavy Chain 11 (*DNAH11*), and Cilia and Flagella Associated Protein 300 (*CFAP300*). These mutations cause defective sperm motility and chronic respiratory disease due to impaired ciliary motility [23,24,25]. Whole-exome sequencing has facilitated the discovery of rare, family-specific mutations that account for subtle sperm motility phenotypes such as multiple morphological abnormalities of the sperm flagella (MMAF) [26,27]. ICSI remains a highly successful treatment, even though there is always the danger of transmission of disease-causing variants if the disorder has an autosomal recessive inheritance. Genetic diagnosis allows for personalized counselling and targeted therapy [28,29,30].

#### 2.2.3. Endocrine and Neurologic Genetic Disorders

Primary and secondary hypogonadism are caused by genetic disorders of gonadotropin signalling, androgen sensitivity, or hypothalamic–pituitary development. Mutations of Anosmin 1 (*KAL1*) and Fibroblast Growth Factor Receptor 1 (*FGFR1*) lead to Kallmann syndrome, and mutations of the *GNRHR* and *FSHR* genes can result in isolated hypogonadotropic hypogonadism. Mutations of the androgen receptor (AR) gene result in varied entities of androgen insensitivity syndrome (AIS), from subtle spermatogenic failure to complete testicular feminization. Cytochrome P450 Family 21 Subfamily A Member 2 (*CYP21A2*) and Steroid 5 Alpha-Reductase 2 (*SRD5A2*) defects affect steroid biosynthesis and 5α-reductase activity. These disorders highlight the intersection of endocrinology and genetics in male infertility, with the necessity of molecular evaluation before ART [31,32].

#### 2.2.4. Neuromuscular Repeat Expansion Disorders

Trinucleotide repeat expansion disorders such as Kennedy’s disease (spinal bulbar muscular atrophy; AR gene CAG expansion) and myotonic dystrophy type 1 (Dystrophia Myotonica Protein Kinase—*DMPK* gene CTG expansion) are increasingly being recognized as etiologies of subfertility. These illnesses have spermatogenic failure and may transmit unstable repeat expansions to offspring through ART. Ethical considerations and genetic counseling are crucial because affected males may transmit neurodegenerative or systemic illness. Preimplantation genetic testing for monogenic disorders (PGT-M) is strongly indicated for such couples [33,34].

### 2.3. Chromosomal Abnormalities

Chromosomal abnormalities underlie 5–10% of the causes of genetic male infertility, with sex chromosome aneuploidies being common. The most common karyotypic abnormality is Klinefelter syndrome (47,XXY), seen in approximately 10% of azoospermic men. In mosaic forms (46,XY/47,XXY), restricted spermatogenesis is often preserved, and viable sperm can be recovered for ICSI. Although children of Klinefelter patients conceived by ICSI are usually chromosomally normal, preimplantation genetic testing (PGT-A) is recommended to prevent the transmission of aneuploid embryos. On the other hand, 47,XYY syndrome is generally associated with a milder spermatogenic defect but a larger proportion of disomic spermatozoa. The application of fluorescence in situ hybridization (FISH) and single-cell genomic analysis has also enhanced risk assessment for chromosomal transmission by ART [35,36,37].

Structural chromosome rearrangements, including reciprocal and Robertsonian translocations, inversions, and deletions, can disrupt meiotic segregation and cause infertility. Balanced translocations occur in approximately 1% of infertile men—10 times the frequency in the normal population. High-resolution breakpoint mapping and identification of disrupted genes are now possible with current cytogenetic and sequencing techniques. Couples carrying balanced translocations benefit from PGT-SR (structural rearrangement testing) in preventing the implantation of unbalanced embryos [38,39,40].

### 2.4. Y-Chromosome Microdeletions

Microdeletions in the azoospermia factor (AZF) regions of the Y chromosome are a major cause of non-obstructive azoospermia and severe oligozoospermia. The AZF locus, which spans AZFa, AZFb, and AZFc, contains a number of gene families that are important for spermatogenesis, including *USP9Y*, *DBY*, *RBMY*, *DAZ*, and *CDY1*. Complete deletions of AZFa or AZFb result in Sertoli cell-only syndrome or spermatogenic arrest, while deletions of AZFc have variable phenotypes ranging from hypospermatogenesis to azoospermia. Current research has now also described partial deletions such as gr/gr and b2/b3, with variable effects depending on the Y haplogroup background. Men with AZFc deletions can, on the odd occasion, produce viable sperm and successful ICSI can be obtained, but all male offspring will inherit the same deletion, transmitting infertility through generations [41,42,43].

Technology has enhanced detection from PCR-based assays to high-throughput sequencing and digital droplet PCR, enabling more accurate delineation of the boundaries of microdeletions and structural polymorphisms. Guidelines now suggest Y-chromosome microdeletion testing in all men with sperm concentrations below 5 million/mL. Cryopreservation of sperm from AZFc-deleted patients is advised due to ongoing testicular degeneration. Genetic counseling should explicitly address the certainty of transmission to the male offspring and potential associations with chromosomal instability and aneuploid risk [44].

### 2.5. Sperm Chromosomal Aneuploidy and Genetic Instability

Sperm from infertile men carry significantly greater frequencies of chromosomal aneuploidy than sperm from fertile controls [45]. Those who have oligozoospermia, testicular failure, or chromosomal rearrangements have increased disomy for sex chromosomes and autosomes 13, 18, and 21 [46]. ICSI with these sperm may result in embryos with mosaicism or aneuploidy that may be detected by preimplantation genetic diagnosis. Single-cell high-throughput sequencing has confirmed that the degree of sperm aneuploidy correlates with the severity of impairment of spermatogenesis [47]. These findings emphasize the need for gamete genetic testing in ART to minimize the transmission of chromosomal abnormalities [46].

## 3. Epigenetic Control and Dangers in Assisted Reproductive Technologies

### 3.1. Epigenetic Reprogramming in the Male Germ Line

Spermatogenesis entails extensive epigenetic remodeling that ensures correct transmission of paternal genomic information while re-establishing epigenetic marks for embryonic development. During spermatogonial differentiation, histones are replaced sequentially by transition proteins and protamines, condensing the paternal genome and establishing a highly specialized chromatin state. Concurrently, DNA methylation patterns and histone modifications such as H3K4me3 and H3K27me3 coordinate gene silencing and activation. Defects in this exquisitely coordinated process undermine sperm function and embryonic developmental potential [8]. Defects in DNA methyltransferases (DNMT1, DNMT3A/B) or histone-modifying enzymes have been associated with spermatogenic arrest, defective chromatin packaging, and impaired fertilizing ability [8]. The dynamic nature of these epigenetic marks makes them vulnerable to environmental, metabolic, and age insults, with the potential to cause heritable epimutations [8,48].

### 3.2. Environmental and Lifestyle Impacts on the Sperm Epigenome

Emerging evidence indicates that environmental exposures can reshape the sperm epigenome and influence offspring health. Smoking, obesity, endocrine-disrupting chemicals, air pollution, and advanced paternal age have been associated with abnormal DNA methylation and histone retention in spermatozoa. Oxidative stress, which represents a common final denominator for many of these exposures, results in base oxidation, protamination disruption, and imprinted gene methylation disturbance at loci such as H19 and Insulin-like Growth Factor 2 (IGF2). Folate, zinc, and omega-3 fatty acids represent some of the nutritional influences that impact one-carbon metabolism and can be utilized to buffer epigenetic defects partially. Animal models demonstrate that high-fat or low-protein paternal diets reprogram sperm small-RNAs content, transmitting metabolic phenotypes to subsequent generations. The results support the concept that pre-conceptional paternal lifestyle contributes to the epigenetic quality of gametes and may influence ART outcomes [49,50].

### 3.3. ART-Induced Epigenetic Perturbations

While ART procedures have been highly successful, they involve non-physiological manipulations, which may interfere with natural epigenetic reprogramming. Ovarian hyperstimulation, in vitro gamete manipulation, composition of the culture medium, cryopreservation, and micromanipulation have all been involved in subtle epigenetic defects [51]. ART-conceived versus naturally conceived offspring comparisons reveal minor but significant differences in DNA methylation status, particularly at imprinted genes. The incidence of imprinting disorders such as Beckwith–Wiedemann, Silver–Russell, and Angelman syndromes is slightly increased in children conceived by ICSI or in vitro fertilization, which suggests that epigenetic remodeling in early embryos is ex vivo condition-sensitive [52,53,54]. These effects, nevertheless, appear to be stochastic rather than deterministic and are influenced by the embryo culture duration, oxygen tension, and parental epigenetic status [55,56].

### 3.4. Genomic Imprinting and Parent-of-Origin Effects

Genomic imprinting ensures parent-specific monoallelic expression of key developmental genes, and its failure results in congenital malformations and abnormal growth patterns. Methylation imprints in the male germ line are established during fetal and perinatal life and maintained throughout spermatogenesis. ART bypasses natural mechanisms of sperm selection for epigenetically stable gametes, and concern exists that the zygote will inherit flawed imprinting. Aberrant methylation of *H19*, Potassium Voltage-Gated Channel Subfamily Q Member 1 Opposite Strand/Antisense Transcript 1 (*KCNQ1OT1*), and Maternally Expressed 3 (*MEG3*) has been described in infertile men’s sperm and in embryos from ICSI cycles. These imprinting alterations can also be found in somatic tissues of the offspring, pointing to the transgenerational effect of paternal epigenetic integrity. Despite the generally low risk, clinical vigilance and long-term follow-up studies are warranted [57,58].

### 3.5. Sperm Small RNAs and Transgenerational Inheritance

Along with DNA and histone modifications, spermatozoa carry a sophisticated inventory of small noncoding RNAs—including microRNAs (miRNAs), PIWI-interacting RNAs (piRNAs), and tRNA-derived fragments (tsRNAs)—that contribute to early embryonic gene regulation. The RNA cargo can be reconfigured in response to environmental or metabolic insults, thereby reprogramming embryonic transcriptional programs after fertilization. Experimental models have shown that fathers’ stress, diet, and toxin exposure can reprogram offspring metabolism and behaviour via sperm-borne small RNAs, without changing DNA sequence. These findings have redefined paradigms of inheritance, positioning the sperm epigenome as a vector of environmental memory. Although emerging human studies suggest an association between sperm small RNA signatures and fertility potential or ART outcomes, the available evidence remains limited and exploratory, and these signatures have not yet been validated for routine clinical use [11,59].

### 3.6. Interaction Between Epigenetic and Genetic Mechanisms

Genetic and epigenetic mechanisms interact strongly in the pathophysiology of male infertility. Mutations in genes that regulate chromatin remodeling, such as Tudor Domain Containing 9 (*TDRD9*), *DNMT3L*, and Lysine Acetyltransferase 8 (*KAT8*), result in secondary epigenetic disruption, while epigenetic instability can predispose to genomic damage and aneuploidy. Oxidative DNA damage and defective DNA repair generate both sequence mutation and abnormal methylation, establishing a vicious cycle of genomic and epigenomic instability. In ART, these mechanisms can converge: paternal germline defects predispose to culture-induced epimutations, and in vitro stress exacerbates the expression of occult genetic susceptibilities. Combined multi-omics approaches now permit simultaneous assessment of sperm DNA integrity, chromatin accessibility, and methylation, with unprecedented resolution of this interplay [6,10].

### 3.7. Clinical Implications and Risk Mitigation

Detection of epigenetic susceptibility in ART has prompted laboratory protocol optimization to minimize environmental stress during gamete and embryo handling. Optimized oxygen concentrations (5%), antioxidant supplementation, and shorter durations of in vitro culture have been shown to preserve normal methylation patterns [60]. Epigenetic testing of sperm prospectively—through methylation arrays or small-RNA profiling—is being refined as an adjunct to conventional semen analysis. In addition, preconception lifestyle modification and antioxidant therapy can improve sperm epigenetic stability [49]. Preconceptional counselling of couples regarding these factors is a feasible strategy for reducing the epigenetic burden transmitted through ART [61] (Table 1).

## 4. Molecular Mechanisms Bridging Genetics and Epigenetics in Male Infertility

### 4.1. Oxidative Stress as a Common Pathogenic Driver

Oxidative stress is perhaps the most common mechanism coupling genetic and epigenetic damage in the male germ line. Reactive oxygen species (ROS) generated following inflammation, testicular heat stress, or environmental exposures target sperm DNA, lipids, and proteins. With mature spermatozoa possessing minimal antioxidant defences and lacking DNA repair capability, oxidative lesions such as 8-hydroxy-2′-deoxyguanosine (8-OHdG) accumulate with high velocity, causing single- and double-strand breaks. These lesions cause mutations, chromosomal rearrangements, and aberrant methylation of CpG islands. ROS also oxidize methylcytosine to hydroxymethylcytosine, interfering with DNA methyltransferase binding and leading to epigenetic drift. The occurrence of elevated oxidative markers in seminal plasma is strongly associated with sperm DNA fragmentation, global hypomethylation, and decreased fertilization rates following ICSI. Cumulatively, these findings position oxidative stress as a molecular bridge between genotoxic and epigenetic instability in male infertility, which can be further modulated by defects in chromatin packaging (see Section 4.2) [62,63].

### 4.2. DNA Damage Response and Chromatin Remodeling

The histone-to-protamine-based chromatin remodelling in spermiogenesis entails the precise coordination of topoisomerases, nucleases, and chromatin-remodelling enzymes. Disruption of this process is manifested by incomplete protamination, residual nucleosomal retention, and an open chromatin structure prone to DNA breaks. Mutations or dysregulation of histone acetyltransferases (*KAT8*, cAMP Responsive Element Binding Lysine Acetyltransferase—*CREBBP*), deacetylases (Histone Deacetylase 1—*HDAC1*, Sirtuin 1—*SIRT1*), and ATP-dependent remodelers (SWI/SNF Related BAF Chromatin Remodeling Complex Subunit ATPase 4—*SMARCA4*, Chromodomain Helicase DNA Binding Protein 5—*CHD5*) have been associated with faulty sperm chromatin packaging. Abnormal protamine P1/P2 ratios are always associated with increased DNA fragmentation and aberrant methylation of paternally imprinted loci. Under ART, spermatozoa bearing such chromatin defects are still fertilizing but may transmit DNA lesions not repaired after fertilization, resulting in embryo arrest or de novo mutations. Chromatin remodelling defects thus constitute a molecular interface through which epigenetic deregulation is translated into genetic instability [64,65].

### 4.3. Sperm Energy Metabolism and Mitochondrial Dysfunction

Mitochondria are important determinants of sperm motility and redox balance. Accumulation of mtDNA mutations, either inherited or developed as a result of oxidative damage, derails ATP synthesis and enhances ROS production. Sperm with greater mtDNA copy number and deletions have compromised motility and fertilizing capacity. Furthermore, mtDNA mutations can indirectly influence nuclear epigenetics by virtue of altered NAD^+^/NADH ratios and modulation of one-carbon metabolism, thereby controlling histone and DNA methylation patterns. Recent metabolomic studies demonstrate that mitochondrial dysfunction accounts for global sperm hypomethylation and defective imprint establishment, and mitochondrial–epigenetic cross-talk is implicated in the core of male infertility and ART success [66,67].

### 4.4. DNA Methylation Drift and Epimutation Propagation

Male germ cells undergo sequential waves of de- and re-methylation during development to establish sex-specific imprinting and maintain genomic stability. Aberrant methylation patterns, either due to mutations in methyltransferases (*DNMT3A*, *DNMT3L*) or environmental stress, cause localized or global hypomethylation. This destabilizes repetitive elements, promoting transposon activation and double-strand breaks. Methylation drift stochastically accrues across spermatogenic generations, predisposing individuals to meiotic errors and sperm aneuploidy with time. In ART, the use of sperm from older men or those experiencing chronic oxidative stress exacerbates such epigenetic erosion, with the possibility of inducing imprinting instability in embryos. The perseverance of such methylation errors into offspring somatic tissues underscores their heritability [68].

### 4.5. Dysregulation of Noncoding RNA and Post-Transcriptional Regulation

Testicular epigenetic regulation is not confined to chromatin marks but also includes small and long noncoding RNAs (ncRNAs). These molecules govern transcriptional activity and genome protection during spermatogenesis. piRNAs, for instance, silence transposable elements, while tRNA-derived fragments modulate translation efficiency. Mutations in piRNA biogenesis genes (Piwi Like RNA-Mediated Gene Silencing 1—*PIWIL1*, *TDRD9*, Mov10 Like RNA helicase 1—*MOV10L1*) cause failure of spermatogenesis with widespread transposon de-repression and epigenetic collapse. Environmental insults and ART conditions can alter sperm small-RNA content, reshaping early embryonic transcriptomes and developmental trajectories. This intersection of RNA-mediated and chromatin-based control is a hallmark of how genetic and epigenetic pathways interlock to safeguard germline integrity [69].

### 4.6. Oxidative-Epigenetic Feedback Loops in ART

The ART laboratory environment can intensify oxidative–epigenetic interactions. Sperm centrifugation, prolonged incubation, and cryopreservation procedures induce ROS and disrupt sperm nuclear architecture. Oxidized guanine residues within promoter CpG islands compromise methylation maintenance, inducing localized epimutations. Conversely, aberrant methylation of antioxidant defence genes such as Superoxide Dismutase 2 (*SOD2*) and Glutathione Peroxidase 4 (*GPX4*) further reduces the tolerance of the sperm to oxidative stress, generating a self-sustaining cycle. Clinical evidence indicates that men with high sperm DNA fragmentation and aberrant methylation patterns have lower blastocyst formation rates and increased early miscarriage following ICSI. Antioxidant-containing media and optimized oxygen tension (5%) have been shown to buffer such effects, emphasizing the need for molecularly driven ART optimization [70].

### 4.7. Multi-Omics Integration and Systems-Level Insights

Emerging multi-omics technologies have enabled the in-depth profiling of the sperm epigenome, transcriptome, proteome, and metabolome. Integrative investigations demonstrate that infertile men exhibit a concerted disruption across these molecular layers, reflecting convergent pathophysiologic processes. For example, hypomethylation of chromatin remodeler promoters (CHD5, Bromodomain Testis Associated—BRDT) is linked to altered histone retention and downstream proteomic defects. Such multi-level derangement suggests that male infertility is not a failure of a single molecular process but a systems failure of genome maintenance. These findings form the basis of a paradigm shift towards personalized, molecularly driven reproductive medicine, with sperm “omics” signatures guiding diagnosis, prognosis, and ART strategy [71,72] (Table 2).

## 5. Clinical and Ethical Implications in ART

### 5.1. Integration of Molecular Diagnostics in Infertility Investigation

It has been recognized that epigenetic and genetic abnormalities are a significant aetiology of male infertility, and this has transformed diagnostic paradigms. Traditional semen analysis, while helpful in assessing sperm count, motility, and morphology, will not detect molecular abnormalities underlying possible negative reproductive outcomes. The incorporation of advanced diagnostics—karyotyping, Y-chromosome microdeletion analysis, *CFTR* mutation screening, and next-generation sequencing panels for targeted genes—has become the norm in tertiary fertility centres. Most recently, epigenetic profiling tests, including sperm DNA methylation arrays and small-RNA signatures, have emerged as highly promising biomarkers of sperm quality and embryo developmental potential [17,73]. However, these assays are still experimental, require further standardization and validation, and their utility for clinical diagnosis or counselling remains to be established.

### 5.2. Preimplantation Genetic and Epigenetic Testing

Preimplantation genetic testing (PGT) is a cornerstone of risk reduction in ART for those with known genetic disease. PGT for aneuploidy (PGT-A) improves implantation efficiency and reduces miscarriage risk by selecting euploid embryos. PGT for monogenic disorders (PGT-M) allows the detection of single-gene defects such as *CFTR* mutations or repeat expansions, while PGT for structural rearrangements (PGT-SR) is applicable in translocation carriers. Recent developments in genome-wide haplotyping and low-input sequencing enable the simultaneous identification of chromosomal and point mutations from small embryo biopsy samples. Although standard epigenetic testing is not yet routinized, pilot studies using bisulfite sequencing and methylation arrays show potential for identifying embryos with abnormal imprinting or methylation instability. These integrated approaches herald a new age of precision embryology in which genomic and epigenomic integrity drive embryo choice [74,75].

### 5.3. Genetic Counselling and Risk Communication

Genetic counselling is necessary for the explanation of test results, transmission risk discussion, and managing ethical concerns in ART. Men who have Y-chromosome microdeletions, for instance, must be informed that any male child born by ICSI will also inherit the deletion and may be infertile. Similarly, couples where both partners are carriers of a *CFTR* mutation or chromosomal rearrangement must be counselled about the potential need for PGT or donor gametes. Counselling needs to move beyond deterministic risk to cover probabilistic and epigenetic influences, as well as the possible but immeasurable risk of imprinting disorders. Increasing molecular complexity demands multidisciplinary teams—clinical geneticists, reproductive endocrinologists, and bioethicists—to promote open and even-handed communication with patients [76,77].

### 5.4. Sperm Selection and Processing Advances

On the basis of the proven interrelationships between sperm DNA fragmentation, chromatin instability, and ART outcomes, new sperm selection methods have been developed to identify gametes with improved molecular integrity. Hyaluronic acid (HA) binding assays, magnetic-activated cell sorting (MACS), and microfluidic sperm sorting systems are a few of the methods that exploit biophysical and biochemical markers of sperm maturity and DNA integrity [78,79]. Raman spectroscopy and micro-optical tweezers are being investigated for non-invasive molecular screening of spermatozoa before ICSI. These technologies can enrich for sperm with intact chromatin and healthy epigenetic marks, leading to improved fertilization and embryo quality. Although still experimental, their integration into ART workflows can be anticipated to reduce the burden of genetic and epigenetic defects transmitted to offspring [80,81].

### 5.5. Lifestyle, Nutritional, and Pharmacologic Interventions

The epigenome of sperm is liable to dynamic alteration, and its stability could be reinforced through counteractive measures against oxidative and metabolic stress. Lifestyle modification (i.e., smoking, weight, and alcohol consumption reduction) optimises semen parameters and reduces DNA fragmentation. Nutritional therapy with antioxidants (vitamin C, vitamin E, coenzyme Q10, selenium, zinc) and methyl donors (folate, betaine) supports one-carbon metabolism and methylation homeostasis. Novel pharmacologic therapies targeting mitochondrial activity and redox status are also promising to restore sperm epigenetic integrity. Randomized trials have demonstrated that preconception antioxidant treatment improves fertilization and live birth rates in couples undergoing ART, likely through reduction in oxidative DNA damage and normalization of imprinted gene methylation [82].

### 5.6. Offspring Outcomes and Long-Term Follow-Up

Concerns about the health of ART offspring have motivated epigenomic and epidemiologic research on a grand scale over the past two decades. While most ART children experience normal growth and mental development, there has been a small increase in imprinting disorders and cardiometabolic risk factors. Methylation profiling of cord blood and placental tissue discloses subtle but consistent differences in epigenetic marks, namely at imprinted gene clusters and developmental genes. Long-term follow-up studies are beginning to establish whether these differences persist into adulthood or predispose to susceptibility to disease in later life. Current evidence suggests that while ART does not carry a major health risk, continued monitoring of epigenetic outcomes is crucial to intergenerational safety [83].

### 5.7. Ethical and Regulatory Considerations

The potential of ART to circumvent natural selection raises ethical and societal questions regarding germline integrity and the boundaries of medical intervention. Ethical considerations are focused on the transmission risk of genetic or epigenetic defects to subsequent generations and the role of the clinician in minimizing such risks. International guidelines by ESHRE and ASRM emphasize informed consent, disclosure of results of genetic tests, and the avoidance of experimental methods in the absence of satisfactory safety data.

The introduction of genome editing technologies, such as CRISPR-Cas9, has only intensified ethical debate, particularly for the hypothetical correction of inherited mutations in embryos or gametes. However, beyond ethical objections, substantial legal and social barriers currently prevent their reproductive applications. Many countries have explicit legislative prohibitions on germline modification (such as those embedded in the Oviedo Convention and national reproductive medicine acts) while others enforce de facto bans through regulatory agencies that restrict the creation or clinical use of gene-edited embryos. Artificial or in vitro-derived gametes face similar regulatory constraints, including requirements for extensive preclinical safety data and unresolved questions surrounding lineage assignment, donor status, and parental rights. Reflecting these ongoing concerns, in 2023, a London-based international consortium of experts reaffirmed at the Third International Summit on Human Genome Editing that heritable human genome editing remains unacceptable for clinical use at this time, emphasizing the need for more evidence on safety and efficacy and for broader societal discussion, even if full consensus is not required by local authorities [84]. Continued debate among researchers, ethicists, regulators, and the public is still required, not only to balance reproduction autonomy with genomic stewardship in the long term but also to determine whether and under what conditions such technologies could ever be responsibly integrated into reproductive practice [85,86].

### 5.8. Future Directions in Clinical Translation

The advent of artificial intelligence and multi-omics diagnostics in reproductive medicine has created new opportunities for personalized medicine. Machine learning algorithms have the potential to integrate genetic, epigenetic, and phenotypic datasets for the prediction of ART outcomes and embryo selection. Single-cell sequencing and spatial transcriptomics will offer a glimpse into gamete and embryo development at a more profound level, guiding individualized intervention strategies. Meanwhile, ethical standards must evolve to address data privacy, algorithmic bias, and equitable access to emerging technologies. The confluence of molecular biology, computer science, and bioethics will form the future of precision reproductive medicine, leading ART from empirical to data-driven science [87,88].

## 6. Future Perspectives

### 6.1. The Era of Multi-Omics and Systems Reproductive Biology

The integration of genomics, epigenomics, transcriptomics, proteomics, and metabolomics is reshaping the field of male infertility research. Multi-omics investigations reveal that sperm pathology does not result from a single molecular defect but is an expression of systemic dysregulation at the biological strata levels. Concurrent DNA methylation, chromatin accessibility, and RNA expression profiling is feasible now and allows for the identification of molecular signatures for sperm quality prediction and ART outcome. Recent studies with single-cell RNA sequencing have characterized transcriptional heterogeneity among spermatogonial and Sertoli cell populations and revealed new candidate genes involved in spermatogenic arrest. Systems biology approaches linking omics data to clinical phenotypes are at the forefront of developing integrative diagnostic models that can personalize infertility treatment and simplify ART protocols [1,6].

### 6.2. Artificial Gametogenesis and In Vitro Spermatogenesis

One of the active frontiers in reproductive biology is the differentiation of functional gametes from stem cells. The advent of induced pluripotent stem cell (iPSC) technology and organoid culture systems has made it possible to conduct in vitro spermatogenesis with increasing feasibility [89]. These systems recapitulate key stages of germ cell differentiation, epigenetic reprogramming, and meiosis, providing experimental models for the investigation of infertility mechanisms and testing of therapeutic interventions. Artificial gametogenesis can potentially be useful for those with complete germline failure, i.e., AZFa/b deletions or gonadotoxic damage. However, the establishment of appropriate imprinting and chromatin structure is a major hurdle. Preclinical data show that in vitro-derived gametes exhibit aberrant methylation at imprinted loci, emphasizing the importance of stringent epigenetic testing before clinical application. Ethical and regulatory frameworks must evolve alongside to govern the responsible use of such technologies [90].

### 6.3. CRISPR-Based Functional Genomics and Gene Correction

Genome editing tools such as CRISPR-Cas9, base editors, and prime editors have emerged as integral to elucidating gene function in spermatogenesis and infertility. Precise correction of *CFTR*, *TEX11*, and *SYCP3* mutations in animal models has reinstated fertility, showcasing therapeutic promise. In human germline research, however, stringent ethical restrictions prevent reproductive applications. Even so, CRISPR-based functional genomics enables modelling of patient-derived mutations in vitro with unprecedented molecular mechanism resolution. The emergence of high-fidelity and epigenome-targeting CRISPR variants can one day enable reprogramming of aberrant sperm epigenomes with precision or rescue of developmentally arrested spermatogenesis without altering the DNA sequence. These technologies are slated to be utilized exclusively in research environments for the foreseeable future but do mark the convergence of mechanistic understanding and translational potential within reproductive genetics [91].

### 6.4. Artificial Intelligence in Reproductive Genomics

Artificial intelligence (AI) and machine learning (ML) will be increasingly applied to high-dimensional biological data analysis and ART outcome prediction for some time to come. Deep learning models trained on sperm imagery, embryo morphokinetics, and molecular profiles have the ability to recognize subtle patterns that are not visible to the human eye. The combination of genomic and epigenomic information with AI-based decision systems allows for embryo selection and personalized stimulation protocols to be more objective. Predictive models using sperm methylation patterns and fragmentation indices are being established to predict fertilization potential and implantation success. With improved computational precision, AI systems will find a place in ART laboratories, complementing the abilities of embryologists and geneticists. Transparency of algorithms and data ethics will be crucial in ensuring fair and unbiased application of such technologies [92].

### 6.5. Epigenetic Therapies and Reproductive Precision Medicine

A novel goal in reproductive medicine is the correction or stabilization of epigenetic defects before conception. Epigenetic therapies—ranging from small-molecule modulators of DNMTs and HDACs and dietary interventions to antioxidant supplementation—are being investigated to restore sperm epigenome integrity. Pharmacologic agents that enhance NAD^+^ metabolism and sirtuin activity have demonstrated potential in optimizing chromatin compaction and mitochondrial function. The pre-conceptional window is a critical period for such therapies since sperm epigenetic marks are continuously remodelled during spermatogenesis. The future model is likely to involve molecular stratification of patients according to omics-based biomarkers, then targeted lifestyle and pharmacologic strategies to optimize germline health prior to ART [12].

### 6.6. Transgenerational Health and Longitudinal Cohorts

Ascertaining the long-term impact of ART on offspring health remains an active research area. Large-scale, multi-generational cohort studies are required to assess subtle genetic and epigenetic impacts over decades. Integration of biobank data, electronic health records, and molecular profiling will enable follow-up of developmental, metabolic, and neurocognitive outcomes in ART-conceived children. Recent evidence suggests that certain epigenetic alterations induced by ART are reversible or compensated post-natally, highlighting the plasticity of the epigenome. Longitudinal studies should therefore seek to identify permanent versus transient changes and their correlation with diseases of adult onset. These data will inform future ART optimization for reproductive success without intergenerational health compromise [93,94].

### 6.7. The Path Toward Molecularly Guided Reproduction

The introduction of molecular data into clinical decision-making marks a new era for reproductive medicine. Global preconception evaluation of the male germline (integrating genetic, epigenetic, and environmental considerations) will guide individualized ART strategies. AI-driven analysis of omics data will assist clinicians in a trade-off between reproductive effectiveness and genomic protection. Future ART programs will incorporate molecular diagnostics, predictive modelling, and precision intervention to establish a closed-loop system of “molecularly guided reproduction.” Such development will require collaboration among geneticists, embryologists, data scientists, and ethicists and foster a framework that maximizes reproductive freedom while guarding against heritable genomic integrity [95].

## 7. Conclusions

During the last two decades, genetic and epigenetic research advances have revolutionized the field of male infertility and its relevance to assisted reproduction. What was previously considered to be largely endocrine or idiopathic is now considered a multifactorial process with complex interactions between genomic integrity, chromatin structure, and environmental regulation. Single-gene defects, Y-chromosome deletions, chromosomal abnormalities, and copy-number variations are coupled with aberrant methylation, disrupted histone retention, and altered small-RNA content.

Widespread use of ICSI and other ART modalities has enabled fatherhood in men carrying severe spermatogenic defects. However, such technological success conveys to ART the potential to inadvertently pass on genetic or epigenetic flaws to the next generation. While the absolute risk for disorders is low, molecular abnormalities have been described in ART offspring. These reports stress that the integration of molecular diagnostics, preimplantation testing, and long-term epidemiologic surveillance is needed in reproductive medicine.

Artificial intelligence may assist practitioners in interpreting complex genomic and epigenomic data, ART outcome prediction, and the determination of the best embryos for transfer. The future of reproductive medicine will be balancing technological capability with ethical responsibility, so that the pursuit of fertility is not at the cost of undermining the integrity of the human germline.

## Figures and Tables

**Table 1:** Epigenetic Control and Dangers in Assisted Reproductive Technologies.

Mechanism/Theme	Key Processes and Players	Molecular Consequences	Epigenetic/Genetic Links	Clinical/ART Implications
Epigenetic reprogramming in the male germ line	Histone → transition proteins → protamines: DNMT1, DNMT3A/B; histone marks (H3K4me3, H3K27me3)	Defective chromatin condensation and spermatogenic arrest	Aberrant DNA methylation, histone modification loss, heritable epimutations	Reduced sperm quality and fertilizing ability; age/environment-sensitive vulnerability [8]
Environmental and lifestyle impacts	Smoking, obesity, EDCs, pollution, paternal age; oxidative stress; nutrients (folate, zinc, ω-3)	DNA base oxidation, protamine disruption	Altered CpG methylation at H19/IGF2; histone retention; small-RNA reprogramming	Lifestyle modulates sperm epigenome and ART outcome; nutritional buffering feasible [49,50]
ART-induced epigenetic perturbations	Ovarian stimulation, culture media, cryopreservation, micromanipulation	Subtle imprinting defects; increased oxidative stress	Methylation changes at imprinted loci; culture-dependent remodeling	Slight ↑ incidence of imprinting syndromes (BWS, SRS, AS); dependent on culture & O_2_ [52,55,56]
Genomic imprinting and parent-of-origin effects	*H19*, *KCNQ1OT1*, *MEG3* imprints; fetal and perinatal methylation establishment	Loss of imprint stability; monoallelic-expression failure	Aberrant sperm methylation transmitted to embryos and offspring tissues	Rare but transmissible imprint disorders; need for long-term offspring follow-up [57,58]
Sperm small RNAs and transgenerational inheritance	miRNAs, piRNAs, tRNA-derived fragments; RNA cargo reshaping by stress/diet/toxins	Altered embryonic transcription, metabolism, and behavior	Small-RNA-mediated epigenetic inheritance without DNA change	Sperm RNA signature as biomarkers of fertility and ART success [11,59]
Interaction between epigenetic and genetic mechanisms	*TDRD9*, *DNMT3L*, *KAT8* mutations; oxidative DNA damage; defective repair	Sequence mutation + methylation errors; chromatin instability	Feedback loop between genomic and epigenomic instability	Multi-omics profiling reveals interplay; culture stress unmasks genetic risks [6,10]
Clinical implications and risk mitigation	Oxygen (5%) control; antioxidants; shortened culture; methylation and RNA testing	↓ ROS, preserved methylation, stabilized sperm RNA	Targeted reduction in ART-induced epimutations	Lifestyle optimization, antioxidant therapy, pre-conception counselling recommended [49,60,61]

**Table 2:** Molecular Mechanisms Bridging Genetics and Epigenetics in Male Infertility.

Mechanism/Theme	Key Processes and Players	Genetic Effects	Epigenetic Effects	Clinical/ART Implications
Oxidative stress as a driver	ROS from inflammation, heat, or toxins; poor antioxidant defenses; lesions like 8-OHdG	Single-/double-strand breaks, point mutations, chromosomal rearrangements	CpG hypomethylation; 5mC → 5hmC; DNMT interference; epigenetic drift	High ROS ↔ sperm DNA fragmentation, hypomethylation, ↓ ICSI fertilization [62,63]
DNA damage response and chromatin remodeling	Histone → protamine transition; KAT8, CREBBP, HDAC1, SIRT1, SMARCA4, CHD5; P1/P2 ratio	Open chromatin, unrepaired DNA breaks, embryo arrest or de novo mutations	Incomplete protamination, aberrant imprint methylation	Chromatin-defective sperm fertilize in ART but raise embryonic risk [64,65]
Mitochondrial dysfunction and metabolism	mtDNA mutations/deletions; altered ATP, ROS, NAD^+^/NADH, one-carbon metabolism	Reduced motility and fertilizing capacity	Global hypomethylation, defective imprinting via metabolic–epigenetic crosstalk	Mitochondrial health influences ART success [66,67]
DNA methylation drift and epimutation propagation	Aberrant de-/re-methylation; DNMT3A/3L; aging; environmental stress	Transposon activation, double-strand breaks, meiotic errors, aneuploidy	Global hypomethylation; imprinting instability; heritable methylation errors	Aging and oxidative stress worsen drift, affecting embryos [68]
Noncoding RNA dysregulation	piRNAs (PIWIL1, TDRD9, MOV10L1); tRNA fragments; ncRNAs	Transposon de-repression, spermatogenic failure	Altered sperm RNA cargo; disrupted early embryonic transcriptomes	Environmental/ART effects alter sperm RNA profiles [69]
Oxidative-epigenetic feedback in ART	TDRD9, Centrifugation, incubation, cryo → ROS; SOD2; GPX4 promoter methylation	Local oxidative mutations; persistent DNA lesions	Epimutations from faulty methylation; reduced antioxidant gene expression	High SDF ↔ ↓ blastocysts, ↑ miscarriage; antioxidants and 5% O_2_ help [70]
Multi-omics and systems insights	Integration of methylome, transcriptome, proteome, metabolome; CHD5, BRDT	Genome maintenance failure across multiple layers	Epigenetic derangement in multiple omes	Omics-guided precision medicine for ART [71,72]

## Data Availability

No new data was created or analyzed in this study. Data sharing is not applicable to this article.

## Abbreviations

The following abbreviations are used in this manuscript:

AI	Artificial intelligence
AIS	Androgen insensitivity syndrome
AR	Androgen receptor
ART	Assisted reproductive technologies
AZF	Azoospermia factor
BRDT	Bromodomain Testis Associated
CBAVD	Congenital bilateral absence of the vas deferens
CCDC39	Coiled-Coil Domain 39 Molecular Ruler Complex Subunit
CCDC40	Coiled-Coil Domain 40 Molecular Ruler Complex Subunit
CFAP43	Cilia and Flagella Associated Protein 43
CFAP44	Cilia and Flagella Associated Protein 44
CFAP300	Cilia and Flagella Associated Protein 300
CFTR	Cystic Fibrosis Transmembrane Conductance Regulator
CHD5	Chromodomain Helicase DNA Binding Protein 5
CNVs	Copy number variations
CREBBP	cAMP Responsive Element Binding Lysine Acetyltransferase
CYP21A2	Cytochrome P450 Family 21 Subfamily A Member 2
DMPK	Dystrophia Myotonica Protein Kinase
DNAH1	Dynein Axonemal Heavy Chain 1
DNAH5	Dynein Axonemal Heavy Chain 5
DNAH11	Dynein Axonemal Heavy Chain 11
DNAI1	Dynein Axonemal Intermediate Chain 1
DNMT	DNA Methyltransferase
FGFR1	Fibroblast Growth Factor Receptor 1
FISH	Fluorescence in situ hybridization
GPX4	Glutathione Peroxidase 4
HA	Hyaluronic acid
HDAC1	Histone Deacetylase 1
ICSI	Intracytoplasmic sperm injection
IGF2	Insulin-Like Growth Factor 2
iPSC	Induced pluripotent stem cell
KAL1	Anosmin 1
KAT8	Lysine Acetyltransferase 8
KCNQ1OT1	Potassium Voltage-Gated Channel Subfamily Q Member 1 Opposite Strand/Antisense Transcript 1
MACS	Magnetic-activated cell sorting
MEG3	Maternally Expressed 3
miRNAs	microRNAs
ML	Machine learning
MMAF	Multiple morphological abnormalities of the sperm flagella
MOV10L1	Mov10 Like RNA Helicase 1
ncRNAs	Noncoding RNAs
NGS	Next-generation sequencing
NOA	Non-obstructive azoospermia
PCD	Primary ciliary dyskinesia
PGT	Preimplantation genetic testing
PGT-A	Preimplantation genetic testing for aneuploidy
PGT-M	Preimplantation genetic testing for monogenic disorders
PGT-SR	Preimplantation genetic testing for structural rearrangements
piRNAs	PIWI-interacting RNAs
PIWIL1	Piwi Like RNA-Mediated Gene Silencing 1
PRM2	Protamine 2
ROS	Reactive oxygen species
SIRT1	Sirtuin 1
SMARCA4	SWI/SNF Related BAF Chromatin Remodeling Complex Subunit ATPase 4
SOD2	Superoxide Dismutase 2
SRD5A2	Steroid 5 Alpha-Reductase 2
SYCP3	Synaptonemal Complex Protein 3
TDRD9	Tudor Domain Containing 9
TEX11	Testis Expressed 11
TNP1	Transition Protein 1
tsRNAs	tRNA-derived fragments
8-OHdG	8-hydroxy-2′-deoxyguanosine

## References

[1] Assidi M. (2022). Infertility in Men: Advances towards a Comprehensive and Integrative Strategy for Precision Theranostics. Cells.

[2] Henningsen A.-K.A., Opdahl S., Wennerholm U.-B., Tiitinen A., Rasmussen S., Romundstad L.B., Bergh C., Gissler M., Forman J.L., Pinborg A. (2023). Risk of Congenital Malformations in Live-Born Singletons Conceived after Intracytoplasmic Sperm Injection: A Nordic Study from the CoNARTaS Group. Fertil. Steril..

[3] Sciorio R., Esteves S.C. (2022). Contemporary Use of ICSI and Epigenetic Risks to Future Generations. J. Clin. Med..

[4] Tesarik J. (2025). Lifestyle and Environmental Factors Affecting Male Fertility, Individual Predisposition, Prevention, and Intervention. Int. J. Mol. Sci..

[5] Sudhakar D.V.S., Shah R., Gajbhiye R.K. (2021). Genetics of Male Infertility—Present and Future: A Narrative Review. J. Hum. Reprod. Sci..

[6] Wagner A.O., Turk A., Kunej T. (2023). Towards a Multi-Omics of Male Infertility. World J. Men’s Health.

[7] Huang X.-Y., Sha J.-H. (2011). Proteomics of Spermatogenesis: From Protein Lists to Understanding the Regulation of Male Fertility and Infertility. Asian J. Androl..

[8] Cui Y., Deng J., Zhang Y., Du L., Jiang F., Li C., Chen W., Zhang H., He Z. (2025). Epigenetic Regulation by DNA Methylation, Histone Modifications and Chromatin Remodeling Complexes in Controlling Spermatogenesis and Their Dysfunction with Male Infertility. Cell. Mol. Life Sci..

[9] Siebert-Kuss L.M., Dietrich V., Di Persio S., Bhaskaran J., Stehling M., Cremers J.-F., Sandmann S., Varghese J., Kliesch S., Schlatt S. (2024). Genome-Wide DNA Methylation Changes in Human Spermatogenesis. Am. J. Hum. Genet..

[10] Hosseini M., Khalafiyan A., Zare M., Karimzadeh H., Bahrami B., Hammami B., Kazemi M. (2024). Sperm Epigenetics and Male Infertility: Unraveling the Molecular Puzzle. Hum. Genom..

[11] Yang C., Zeng Q.-X., Liu J.-C., Yeung W.S.-B., Zhang J.V., Duan Y.-G. (2023). Role of Small RNAs Harbored by Sperm in Embryonic Development and Offspring Phenotype. Andrology.

[12] Kaltsas A., Markou E., Kyrgiafini M.-A., Zikopoulos A., Symeonidis E.N., Dimitriadis F., Zachariou A., Sofikitis N., Chrisofos M. (2025). Oxidative-Stress-Mediated Epigenetic Dysregulation in Spermatogenesis: Implications for Male Infertility and Offspring Health. Genes.

[13] Donkin I., Barrès R. (2018). Sperm Epigenetics and Influence of Environmental Factors. Mol. Metab..

[14] Hattori H., Hiura H., Kitamura A., Miyauchi N., Kobayashi N., Takahashi S., Okae H., Kyono K., Kagami M., Ogata T. (2019). Association of Four Imprinting Disorders and ART. Clin. Epigenetics.

[15] James E.R., Tasnim M., Riera-Escamilla A., Vigh-Conrad K., Emery B.R., Conrad D.F., Aston K.I. (2025). Genetic and Epigenetic Landscape of Male Infertility. Trends Genet..

[16] Montjean D., Beaumont M., Natiq A., Louanjli N., Hazout A., Miron P., Liehr T., Cabry R., Ratbi I., Benkhalifa M. (2024). Genome and Epigenome Disorders and Male Infertility: Feedback from 15 Years of Clinical and Research Experience. Genes.

[17] Ikbal Atli E., Yalcintepe S., Atli E., Demir S., Gurkan H. (2024). Next-Generation Sequencing Infertility Panel in Turkey: First Results. Balkan J. Med. Genet..

[18] Gudmundsson S., Singer-Berk M., Watts N.A., Phu W., Goodrich J.K., Solomonson M., Rehm H.L., MacArthur D.G., O’Donnell-Luria A., Genome Aggregation Database Consortium (2022). Variant Interpretation Using Population Databases: Lessons from gnomAD. Hum. Mutat..

[19] Cui X., Wu X., Li Q., Jing X. (2020). Mutations of the Cystic Fibrosis Transmembrane Conductance Regulator Gene in Males with Congenital Bilateral Absence of the Vas Deferens: Reproductive Implications and Genetic Counseling (Review). Mol. Med. Rep..

[20] Sorrentino U., Menegazzo M., Gabbiato I., Calosci D., Zambon C.F., Zuccarello D. (2024). Challenges of Preimplantation Genetic Counselling in the Context of Cystic Fibrosis and Other CFTR-Related Disorders: A Monocentric Experience in a Cohort of 92 Couples. Genes.

[21] Anjankar N., More A., Anjankar A.P., Mahajan S.S., Nawale N. (2025). CFTR Gene Mutations and Their Role in Male Infertility: A Case Study. J. Pharm. Bioallied Sci..

[22] Wosnitzer M.S. (2014). Genetic Evaluation of Male Infertility. Transl. Androl. Urol..

[23] Sironen A., Shoemark A., Patel M., Loebinger M.R., Mitchison H.M. (2020). Sperm Defects in Primary Ciliary Dyskinesia and Related Causes of Male Infertility. Cell. Mol. Life Sci..

[24] Aprea I., Wilken A., Krallmann C., Nöthe-Menchen T., Olbrich H., Loges N.T., Dougherty G.W., Bracht D., Brenker C., Kliesch S. (2023). Pathogenic Gene Variants in CCDC39, CCDC40, RSPH1, RSPH9, HYDIN, and SPEF2 Cause Defects of Sperm Flagella Composition and Male Infertility. Front. Genet..

[25] Yin H.-Y., Zhou Y.-Q., Shen Q.-S., Chen Z.-W., Li J.-R., Wu H., Cao Y.-X., Guo R., Song B. (2025). CFAP300 Loss-of-Function Variant Causes Primary Ciliary Dyskinesia and Male Infertility via Disrupting Sperm Flagellar Assembly and Acrosome Formation. Asian J. Androl..

[26] Xu Y., Yang B., Lei C., Yang D., Ding S., Lu C., Wang L., Guo T., Wang R., Luo H. (2022). Novel Compound Heterozygous Variants in CCDC40 Associated with Primary Ciliary Dyskinesia and Multiple Morphological Abnormalities of the Sperm Flagella. Pharmgenom. Pers. Med..

[27] Chen D., Liang Y., Li J., Zhang X., Zheng R., Wang X., Zhang H., Shen Y. (2021). A Novel CCDC39 Mutation Causes Multiple Morphological Abnormalities of the Flagella in a Primary Ciliary Dyskinesia Patient. Reprod. Biomed. Online.

[28] Castleman V.H., Romio L., Chodhari R., Hirst R.A., de Castro S.C.P., Parker K.A., Ybot-Gonzalez P., Emes R.D., Wilson S.W., Wallis C. (2009). Mutations in Radial Spoke Head Protein Genes RSPH9 and RSPH4A Cause Primary Ciliary Dyskinesia with Central-Microtubular-Pair Abnormalities. Am. J. Hum. Genet..

[29] Gileles-Hillel A., Mor-Shaked H., Shoseyov D., Reiter J., Tsabari R., Hevroni A., Cohen-Cymberknoh M., Amirav I., Brammli-Greenberg S., Horani A. (2020). Whole-Exome Sequencing Accuracy in the Diagnosis of Primary Ciliary Dyskinesia. ERJ Open Res..

[30] Newman L., Chopra J., Dossett C., Shepherd E., Bercusson A., Carroll M., Walker W., Lucas J.S., Cheong Y. (2023). The Impact of Primary Ciliary Dyskinesia on Female and Male Fertility: A Narrative Review. Hum. Reprod. Update.

[31] Hwang K., Yatsenko A.N., Jorgez C.J., Mukherjee S., Nalam R.L., Matzuk M.M., Lamb D.J. (2010). Mendelian Genetics of Male Infertility. Ann. N. Y. Acad. Sci..

[32] Millar A.C., Faghfoury H., Bieniek J.M. (2021). Genetics of Hypogonadotropic Hypogonadism. Transl. Androl. Urol..

[33] Kim W.B., Jeong J.Y., Doo S.W., Yang W.J., Song Y.S., Lee S.R., Park J.W., Kim D.W. (2012). Myotonic Dystrophy Type 1 Presenting as Male Infertility. Korean J. Urol..

[34] Lian M., Lee C.G., Chong S.S. (2019). Robust Preimplantation Genetic Testing Strategy for Myotonic Dystrophy Type 1 by Bidirectional Triplet-Primed Polymerase Chain Reaction Combined with Multi-Microsatellite Haplotyping Following Whole-Genome Amplification. Front. Genet..

[35] Kim I.W., Khadilkar A.C., Ko E.Y., Sabanegh E.S. (2013). 47,XYY Syndrome and Male Infertility. Rev. Urol..

[36] Maiburg M., Repping S., Giltay J. (2012). The Genetic Origin of Klinefelter Syndrome and Its Effect on Spermatogenesis. Fertil. Steril..

[37] Lamb D.J. (2025). Chromosome Defects and Male Factor Infertility. Fertil. Steril..

[38] Verdoni A., Hu J., Surti U., Babcock M., Sheehan E., Clemens M., Drewes S., Walsh L., Clark R., Katari S. (2021). Reproductive Outcomes in Individuals with Chromosomal Reciprocal Translocations. Genet. Med..

[39] Wang H., Jia Z., Mao A., Xu B., Wang S., Wang L., Liu S., Zhang H., Zhang X., Yu T. (2020). Analysis of Balanced Reciprocal Translocations in Patients with Subfertility Using Single-Molecule Optical Mapping. J. Assist. Reprod. Genet..

[40] Shetty S., Nair J., Johnson J., Shetty N., J A.K., Thondehalmath N., Ganesh D., Bhat V.R., M S., R A. (2022). Preimplantation Genetic Testing for Couples with Balanced Chromosomal Rearrangements. J. Reprod. Infertil..

[41] Rabinowitz M.J., Huffman P.J., Haney N.M., Kohn T.P. (2021). Y-Chromosome Microdeletions: A Review of Prevalence, Screening, and Clinical Considerations. Appl. Clin. Genet..

[42] Liu X., Qiao J., Li R., Yan L., Chen L. (2013). Y Chromosome AZFc Microdeletion May Not Affect the Outcomes of ICSI for Infertile Males with Fresh Ejaculated Sperm. J. Assist. Reprod. Genet..

[43] Navarro-Costa P., Plancha C.E., Gonçalves J. (2010). Genetic Dissection of the AZF Regions of the Human Y Chromosome: Thriller or Filler for Male (in)Fertility?. J. Biomed. Biotechnol..

[44] Dai B., Zhao D., Sha R.-N., Cang M. (2025). The Selection of Y Chromosome Microdeletion Detection Methods Based on Seminal Analysis Results: A Comparison of High-Throughput Sequencing and Fluorescence Quantitative Polymerase Chain Reaction (qPCR) Applications. Transl. Androl. Urol..

[45] Luongo F.P., Annunzi E., Girolamo F., Belmonte G., Ponchia R., Piomboni P., Luddi A. (2024). Assessment of Sperm Chromosomal Abnormalities Using Fluorescence in Situ Hybridization (FISH): Implications for Reproductive Potential. J. Assist. Reprod. Genet..

[46] Elnahas R.F., Behery A.K., Kholeif S., Orief Y.I., Elhady G.M. (2023). Sperm Chromosomal Abnormalities in Infertile Men with Failed Intracytoplasmic Sperm Injection (ICSI). Middle East. Fertil. Soc. J..

[47] Sassanarakkit S., Chamnankran S., Singwongsa A., Sukprasert M., Satirapod C. (2024). Chromosomal Analysis of Single Sperm Cells from Infertile Couples with Severe Oligoteratozoospermia: A Cross-Sectional Prospective Study. PLoS ONE.

[48] Ren W., Gao L., Song J. (2018). Structural Basis of DNMT1 and DNMT3A-Mediated DNA Methylation. Genes.

[49] Akhatova A., Jones C., Coward K., Yeste M. (2025). How Do Lifestyle and Environmental Factors Influence the Sperm Epigenome? Effects on Sperm Fertilising Ability, Embryo Development, and Offspring Health. Clin. Epigenetics.

[50] Sudhakaran G., Kesavan D., Kandaswamy K., Guru A., Arockiaraj J. (2024). Unravelling the Epigenetic Impact: Oxidative Stress and Its Role in Male Infertility-Associated Sperm Dysfunction. Reprod. Toxicol..

[51] Sciorio R., Cantatore C., D’Amato G., Smith G.D. (2024). Cryopreservation, Cryoprotectants, and Potential Risk of Epigenetic Alteration. J. Assist. Reprod. Genet..

[52] Ye M., Reyes Palomares A., Iwarsson E., Oberg A.S., Rodriguez-Wallberg K.A. (2024). Imprinting Disorders in Children Conceived with Assisted Reproductive Technology in Sweden. Fertil. Steril..

[53] Cortessis V.K., Azadian M., Buxbaum J., Sanogo F., Song A.Y., Sriprasert I., Wei P.C., Yu J., Chung K., Siegmund K.D. (2018). Comprehensive Meta-Analysis Reveals Association between Multiple Imprinting Disorders and Conception by Assisted Reproductive Technology. J. Assist. Reprod. Genet..

[54] Lazaraviciute G., Kauser M., Bhattacharya S., Haggarty P., Bhattacharya S. (2014). A Systematic Review and Meta-Analysis of DNA Methylation Levels and Imprinting Disorders in Children Conceived by IVF/ICSI Compared with Children Conceived Spontaneously. Hum. Reprod. Update.

[55] Kopca T., Tulay P. (2021). Association of Assisted Reproductive Technology Treatments with Imprinting Disorders. Glob. Med. Genet..

[56] Håberg S.E., Page C.M., Lee Y., Nustad H.E., Magnus M.C., Haftorn K.L., Carlsen E.Ø., Denault W.R.P., Bohlin J., Jugessur A. (2022). DNA Methylation in Newborns Conceived by Assisted Reproductive Technology. Nat. Commun..

[57] Rotondo J.C., Lanzillotti C., Mazziotta C., Tognon M., Martini F. (2021). Epigenetics of Male Infertility: The Role of DNA Methylation. Front. Cell Dev. Biol..

[58] Rahimi S., Shao X., Chan D., Martel J., Bérard A., Fraser W.D., Simon M.-M., Kwan T., Bourque G., Trasler J. (2023). Capturing Sex-Specific and Hypofertility-Linked Effects of Assisted Reproductive Technologies on the Cord Blood DNA Methylome. Clin. Epigenetics.

[59] Liu S., Sharma U. (2023). Sperm RNA Payload: Implications for Intergenerational Epigenetic Inheritance. Int. J. Mol. Sci..

[60] Sciorio R., Rinaudo P. (2023). Culture Conditions in the IVF Laboratory: State of the ART and Possible New Directions. J. Assist. Reprod. Genet..

[61] Menezo Y., Dale B., Elder K. (2018). Time to Re-Evaluate ART Protocols in the Light of Advances in Knowledge about Methylation and Epigenetics: An Opinion Paper. Hum. Fertil..

[62] Aitken R.J. (2020). Impact of Oxidative Stress on Male and Female Germ Cells: Implications for Fertility. Reproduction.

[63] Chakraborty S., Roychoudhury S. (2022). Pathological Roles of Reactive Oxygen Species in Male Reproduction. Adv. Exp. Med. Biol..

[64] Deng T., Xiao Y., Dai Y., Xie L., Li X. (2021). Roles of Key Epigenetic Regulators in the Gene Transcription and Progression of Prostate Cancer. Front. Mol. Biosci..

[65] Hart H.M., Nixon B., Martin J.H., Aitken R.J., De Iuliis G.N. (2025). Improving Sperm Selection Strategies for Assisted Reproduction through Closing the Knowledge Gap in Sperm Maturation Mechanics. Hum. Reprod. Open.

[66] Mai Z., Yang D., Wang D., Zhang J., Zhou Q., Han B., Sun Z. (2024). A Narrative Review of Mitochondrial Dysfunction and Male Infertility. Transl. Androl. Urol..

[67] Vahedi Raad M., Firouzabadi A.M., Tofighi Niaki M., Henkel R., Fesahat F. (2024). The Impact of Mitochondrial Impairments on Sperm Function and Male Fertility: A Systematic Review. Reprod. Biol. Endocrinol..

[68] Zeng Y., Chen T. (2019). DNA Methylation Reprogramming during Mammalian Development. Genes.

[69] Chan S.Y., Wan C.W.T., Law T.Y.S., Chan D.Y.L., Fok E.K.L. (2022). The Sperm Small RNA Transcriptome: Implications beyond Reproductive Disorder. Int. J. Mol. Sci..

[70] Agarwal A., Maldonado Rosas I., Anagnostopoulou C., Cannarella R., Boitrelle F., Munoz L.V., Finelli R., Durairajanayagam D., Henkel R., Saleh R. (2022). Oxidative Stress and Assisted Reproduction: A Comprehensive Review of Its Pathophysiological Role and Strategies for Optimizing Embryo Culture Environment. Antioxidants.

[71] Li W., Wu J., Kim S.-Y., Zhao M., Hearn S.A., Zhang M.Q., Meistrich M.L., Mills A.A. (2014). Chd5 Orchestrates Chromatin Remodelling during Sperm Development. Nat. Commun..

[72] Jin J., Li K., Du Y., Gao F., Wang Z., Li W. (2023). Multi-Omics Study Identifies That PICK1 Deficiency Causes Male Infertility by Inhibiting Vesicle Trafficking in Sertoli Cells. Reprod. Biol. Endocrinol..

[73] Wang C., Swerdloff R.S. (2014). Limitations of Semen Analysis as a Test of Male Fertility and Anticipated Needs from Newer Tests. Fertil. Steril..

[74] Giuliano R., Maione A., Vallefuoco A., Sorrentino U., Zuccarello D. (2023). Preimplantation Genetic Testing for Genetic Diseases: Limits and Review of Current Literature. Genes.

[75] Del Arco de la Paz A., Giménez-Rodríguez C., Selntigia A., Meseguer M., Galliano D. (2024). Advancements and Challenges in Preimplantation Genetic Testing for Aneuploidies: In the Pathway to Non-Invasive Techniques. Genes.

[76] De Die-Smulders C., Van Golde R., Viville S., Sermon K.D. (2023). Genetic Counseling in Assisted Reproductive Treatment. Textbook of Human Reproductive Genetics.

[77] Muthuswamy V. (2011). Ethical Issues in Genetic Counselling with Special Reference to Haemoglobinopathies. Indian J. Med. Res..

[78] Marzano G., Chiriacò M.S., Primiceri E., Dell’Aquila M.E., Ramalho-Santos J., Zara V., Ferramosca A., Maruccio G. (2020). Sperm Selection in Assisted Reproduction: A Review of Established Methods and Cutting-Edge Possibilities. Biotechnol. Adv..

[79] Ribas-Maynou J., Barranco I., Sorolla-Segura M., Llavanera M., Delgado-Bermúdez A., Yeste M. (2022). Advanced Sperm Selection Strategies as a Treatment for Infertile Couples: A Systematic Review. Int. J. Mol. Sci..

[80] Lepine S., McDowell S., Searle L.M., Kroon B., Glujovsky D., Yazdani A. (2019). Advanced Sperm Selection Techniques for Assisted Reproduction. Cochrane Database Syst. Rev..

[81] Pacheco A., Blanco A., Bronet F., Cruz M., García-Fernández J., García-Velasco J.A. (2020). Magnetic-Activated Cell Sorting (MACS): A Useful Sperm-Selection Technique in Cases of High Levels of Sperm DNA Fragmentation. J. Clin. Med..

[82] Skoracka K., Eder P., Łykowska-Szuber L., Dobrowolska A., Krela-Kaźmierczak I. (2020). Diet and Nutritional Factors in Male (In)Fertility-Underestimated Factors. J. Clin. Med..

[83] Hart R.J., Wijs L.A. (2022). The Longer-Term Effects of IVF on Offspring from Childhood to Adolescence. Front. Reprod. Health.

[84] Olson S., Policy and Global Affairs, National Academies of Sciences, Engineering, and Medicine (2023). Third International Summit on Human Genome Editing: Expanding Capabilities, Participation, and Access: Proceedings of a Workshop—In Brief.

[85] Guttinger S. (2018). Trust in Science: CRISPR-Cas9 and the Ban on Human Germline Editing. Sci. Eng. Ethics.

[86] Ayanoğlu F.B., Elçin A.E., Elçin Y.M. (2020). Bioethical Issues in Genome Editing by CRISPR-Cas9 Technology. Turk. J. Biol..

[87] Koplin J.J., Johnston M., Webb A.N.S., Whittaker A., Mills C. (2025). Ethics of Artificial Intelligence in Embryo Assessment: Mapping the Terrain. Hum. Reprod..

[88] Rolfes V., Bittner U., Gerhards H., Krüssel J.-S., Fehm T., Ranisch R., Fangerau H. (2023). Artificial Intelligence in Reproductive Medicine—An Ethical Perspective. Geburtshilfe Frauenheilkd..

[89] Ishii T. (2014). Human iPS Cell-Derived Germ Cells: Current Status and Clinical Potential. J. Clin. Med..

[90] Stewart K.R., Veselovska L., Kelsey G. (2016). Establishment and Functions of DNA Methylation in the Germline. Epigenomics.

[91] Wang H.-Q., Wang T., Gao F., Ren W.-Z. (2022). Application of CRISPR/Cas Technology in Spermatogenesis Research and Male Infertility Treatment. Genes.

[92] More A., Chimurkar V., Mahajan S., Dakre S., Anjankar N., More D. (2025). The Integration of Artificial Intelligence in In Vitro Fertilization: A Comprehensive Narrative Review. J. Pharm. Bioallied Sci..

[93] Ahmadi H., Aghebati-Maleki L., Rashidiani S., Csabai T., Nnaemeka O.B., Szekeres-Bartho J. (2023). Long-Term Effects of ART on the Health of the Offspring. Int. J. Mol. Sci..

[94] Schroeder M., Badini G., Sferruzzi-Perri A.N., Albrecht C. (2022). The Consequences of Assisted Reproduction Technologies on the Offspring Health Throughout Life: A Placental Contribution. Front. Cell Dev. Biol..

[95] Orovou E., Tzimourta K.D., Tzitiridou-Chatzopoulou M., Kakatosi A., Sarantaki A. (2025). Artificial Intelligence in Assisted Reproductive Technology: A New Era in Fertility Treatment. Cureus.

